# Increased **α**-Fetoprotein Predicts Steatosis among Patients with Chronic Hepatitis C Genotype 4

**DOI:** 10.1155/2012/636392

**Published:** 2012-05-14

**Authors:** Nasser Mousa, Yahia Gad, Azza Abdel-Aziz, Ibrahem Abd-Elaal

**Affiliations:** ^1^Department of Tropical Medicine, Mansoura University, Mansoura 35516, Egypt; ^2^Department of Internal Medicine, Mansoura University, Mansoura 35516, Egypt; ^3^Department of Pathology, Mansoura University, Mansoura 35516, Egypt; ^4^Department of Clinical Pathology, Mansoura University, Mansoura 35516, Egypt

## Abstract

*Background*. The prognostic importance of *α*-fetoprotein (AFP) level elevation in patients with chronic hepatitis C and its clinical significance in steatosis associated with HCV infection remain to be determined. The present paper assessed clinical significance of elevated AFP in patients with CHC with and without steatosis. *Methods*. One hundred patients with CHC were divided into 50 patients with CHC and steatosis and 50 patients with CHC and no steatosis based on liver biopsy. *Results*. AFP was significantly increased in CHC with steatosis than patients without steatosis (*P* < 0.001). Highly significant positive correlation was found between serum AFP and necroinflammation as well as the severity of fibrosis/cirrhosis and negative significant correlation with albumin level in chronic HCV with steatosis (*P* < 0.001) but negative nonsignificant correlation with ALT and AST level (*P* ≤ 0.778 and 0.398), respectively. Highly significant increase was found in chronic hepatitis patients with steatosis than CHC without steatosis regarding necroinflammation as well as the severity of fibrosis/cirrhosis and AFP (*P* < 0.001). *Conclusion*. Patients with chronic HCV and steatosis have a higher AFP levels than those without steatosis. In chronic HCV with steatosis, elevated AFP levels correlated positively with HAI and negative significant correlation with albumin level.

## 1. Introduction

Chronic hepatitis C (CHC) is thought to affect more than 170 million people worldwide, and it has been shown that steatosis occurs in approximately 50% of patients with CHC [1]. Steatosis also occurs more than twice as frequently in patients with CHC than in the general population [2]. Both viral and host metabolic factors have been reported to contribute to the genesis of hepatic steatosis in patients with CHC. Most steatosis is mild, with the more severe cases usually occurring in genotype 3 virus infection [3].

Serum alpha-fetoprotein (AFP) is a fetal glycoprotein produced by the yolk sac and fetal liver [4]. Following birth, AFP levels decrease rapidly to less than 20 ng/mL and increase significantly in certain pathologic conditions. Serum AFP is a debated but routinely used marker for hepatocellular carcinoma (HCC) in patients with chronic liver disease [5]. Benign circumstances that may produce elevations of AFP include cirrhosis, hepatic necrosis, acute hepatitis, chronic active hepatitis, ataxia-telangiectasia, and pregnancy [6, 7]. Elevated serum AFP levels may also be due to altered hepatocyte-hepatocyte interaction and the loss of normal architectural arrangements [8]. Based on the results of Ray et al. about 91% of the Egyptian patients with chronic HCV were infected with HCV genotype 4 [9]. In this study, we evaluated serum AFP and its clinical significance in Egyptian patients with chronic hepatitis C mostly genotype 4 (based on the results of Ray et al.) with and without steatosis.

## 2. Subjects and Methods

 This prospective study included 100 patients with CHC divided into two groups (50 patients with steatosis and 50 patients without steatosis) based on liver biopsy. Both groups were adjusting as regarding age, sex, and for risk factors for steatosis (BMI, DM, and hyperlipidemia). They were referred to Mansoura university Hospital, from October 2010 to December 2011, for liver biopsy and searching for chronic HCV management. The study protocol confirmed to the ethical guidelines of 1975 Declaration of Helsinki. Informed consent was obtained from all patients. CHC was defined according to positive serum HCV-Ab and HCV-RNA for at least 6 months and elevated levels of serum aminotransferases (AST, ALT). The patient's medical histories were taken including, age, gender, weight, height, and body mass index (BMI). AFP was studied with ELISA method using Abbott laboratory reagents, USA (normal level of AFP was defined as <8.1 ng/mL); serum fasting triglyceride, fasting blood sugar, albumin, AST, ALT, total bilirubin, and HCV-RNA viral load were determined. Biochemical tests and HCV-RNA (viral load IU/mL) were performed by autoanalyzer (Selecta, Germany) and Cobas Amplicore monitor version 2 (Roche Molecular Systems, Branchburgh, NJ, USA), respectively. BMI was calculated by the following formula: weight (kg)/height² (m²).

 Exclusion criteria were patients with diabetes mellitus, hypertension, and patients who had any serological evidence of infection with other viruses (HBV and HIV); all other known causes of liver diseases were excluded on the basis of analytical, clinical, and epidemiological data: autoimmunity, metabolic and genetic disorders, NASH, alcohol intake, drug toxicity, and patients with decompensated cirrhosis. Hepatocellular carcinoma and other causes of high levels of AFP like cancer of the testes or ovaries and metastatic liver cancer were excluded using ultrasound as the predominant screening method.

 Percutaneous liver biopsy (≥15 mm in length) was performed for all the patients. Liver biopsy specimens were reviewed by a single pathologist. For each liver biopsy specimen, hematoxylin and eosin and Masson's trichrome stains were available. The extent of hepatic steatosis was assessed and graded as none (steatosis < 5%), mild steatosis (steatosis 5–33% of hepatocytes), moderate steatosis (steatosis 34–66% of hepatocytes), and sever (steatosis > 66% of hepatocytes) according to histological scoring system of Kleiner et al. [10]. The histological activity (grade) and degree of fibrosis (stage) of the liver biopsy were assessed according to the modified histological activity index (HAI) of Ishak et al. [11]. Histological activity was considered as minimal (score 1–3), mild (4–8), moderate (9–12), and severe (13–18). Fibrosis was staged separately on a scale 0–6, corresponding to no fibrosis (0), mild (1-2), moderate (3-4), and severe or cirrhosis (5-6).

## 3. Statistical Analysis

The statistical analysis of data was done by using *Excel* program and *SPSS* program (statistical package for social science) version 10. Data are expressed as the mean ± SD. Mean values were compared with the Student's *t*-test (variables with normal distribution) or Mann-Whitney *U*-test (variables with nonnormal distribution). Categorical variables were compared using the chi-square test. Correlations were done using Pearson's correlation. All the tests performed were two sided and a *P* value < 0.05 was considered to be statistically significant.

## 4. Results

This prospective study included 100 patients with CHC (50 patients with steatosis and 50 patients without steatosis) as evidenced by liver biopsy. All patients were positive for anti-HCV antibodies and positive HCV RNA.  Table 1 shows the comparison of clinical, biochemical, and histopathological characteristics of patients with and without steatosis. Patients with steatosis had a significantly higher AFP (*P* < 0.001) and necroinflammation and fibrosis/cirrhosis (*P* < 0.001). Also AST (*P* < 0.001), ALT (*P* = 0.002), total Bilirubin (*P* = 0.023), and prothrombin time (*P* = 0.003) were significant high in chronic HCV with steatosis than without steatosis. However, age, triglyceride, fasting blood glucose, albumin, BMI, and HCV RNA were not significantly different between the two groups.

Table 2 showed a significant positive correlation between higher serum AFP levels, with the severity of periportal necroinflammation, as well as the severity of fibrosis/cirrhosis (*P* < 0.001). A significant negative correlation was found between serum AFP and serum albumin (*P* < 0.001). Also a negative correlation but not significant was found between serum AFP, AST (*P* = 0.398), and ALT (*P* = 0.778).

## 5. Discussion

Hepatitis C virus (HCV) is a member of the Flaviviridae family responsible for acute and chronic liver disease [12]. Infection with HCV is common, with an average worldwide prevalence of 3% [13]. Acute HCV infection becomes persistent in about 85% of cases [14] and may cause chronic hepatitis leading to cirrhosis and, eventually, hepatocellular carcinoma [15].

The reported prevalence of steatosis in patients with chronic hepatitis C varies between 40% and 80%, depending on the features of the population studied in terms of alcohol consumption, prevalence of obesity, diabetes, and other risk factors [16]. The prevalence of steatosis in HCV is approximately 2-fold higher than in another common chronic liver disease like hepatitis B [17], suggesting that HCV may directly cause steatosis, at least in some patients. All genotypes are steatogenic, but numerous reports showed that steatosis was more frequent and more severe in patients infected with genotype 3 [18–20].

AFP is a glycoprotein that is normally generated during conception by the fetal liver and yolk sac. In clinical practice, AFP levels are elevated in various clinical situations, which include hepatocellular carcinoma, acute or chronic viral hepatitis, chronic liver disease, and gonadal tumors [21].

In this study we tried to determine the level of AFP among patients with chronic HCV, and evaluate its relation to the presence of steatosis among 100 patients with chronic HCV (50 with steatosis and 50 without steatosis) enrolled in our study.

Similar to previous studies [6, 7] we found that AFP was increased in patients with chronic HCV in the current study. Although both groups (with or without steatosis) are similar regarding underlying etiology (HCV) and were adjusting for risk factors of high levels of AFP and steatosis, AFP was highly significant among patients with chronic HCV and steatosis than patients without steatosis. Regarding necroinflammmation and fibrosis/cirrhosis, a significant increase in patients with HCV and steatosis than without steatosis was found. Our results were consistent with the previous reports [22, 23] which revealed that HCV is associated with steatosis in a large portion of cases and that steatosis is associated with worsening fibrosis. In addition, steatosis induces chronic hepatic inflammation, reactive oxygen species, and DNA damage in animal models [24–26]. So steatosis is associated with more degree of inflammation and fibrosis. Hepatic progenitor cells (HPCs) arise in the periportal region of the liver and may be responsible for liver regeneration. They express high levels of AFP, certain keratin markers, and GGT [27–29]. Their presence is related to the severity of fibrosis [30], and their activation has been documented in parallel with cells associated with the development of fibrosis (stellate cells) [31]. Since steatosis among patients with chronic HCV infection was associated with an increase in both the number of HPC and the extent of the ductular reaction as provided by Clouston et al., so these provide a potential mechanism whereby steatosis contributes to the increase in AFP [32].

Another explanation of increased AFP was provided by the results of others [27–31]; they concluded that the presence of more fibrosis is associated with increasing number and more activation of hepatic progenitor cells. Since the group with steatosis has more fibrosis/cirrhosis than group without steatosis, hence, the joint association observed in this study among increased AFP in chronic HCV with steatosis than none steatotic group.

The aminotransferases are also important biological markers that are widely used for liver diseases. Elevation of the activity of these enzymes in serum is believed to result from their leakage from damaged cells, and so this reflects hepatocyte injury. These enzymes are elevated in many forms of liver diseases and especially those diseases that are associated with significant hepatocyte necrosis such as acute viral hepatitis, which is the most common cause of massive aminotransferases elevation [33]. In this study the levels of aminotransferases AST and ALT are significantly increased in chronic HCV with steatosis than patients without steatosis, indicating more hepatocytes necrosis in patients with steatosis. Our result was in agreement with Hepburn et al., who found significant increase in ALT and AFP level among patients with steatosis versus without steatosis [34].

Our results showed that serum AFP levels were correlated with the severity of periportal necroinflammation as well as the severity of fibrosis/cirrhosis among chronic HCV with steatosis. These results were consistent with the report of Chu et al., which revealed that higher serum AFP levels were correlated with the severity of periportal necroinflammation as well as the severity of fibrosis/cirrhosis. AFP production is enhanced in the presence of injury, possibly resulting from increased hepatocyte turnover [35]. Hu et al. found a similar correlation between AFP and measures of liver disease activity and severity [36]. The obvious increase in AFP and biochemical values suggests that inflammation, necrosis, and hepatocellular injury are the most common cause of elevated AFP in the studied groups.

In our study a negative correlation was found between lower serum albumin and AFP among patients with steatosis. According to previous reports, the AFP and albumin genes are characteristically arranged in tandem by a similar structure and are believed to be derived from a common ancestral gene [37]. Previous reports have described that reciprocal changes in albumin and AFP gene transcription existed during liver regeneration [38, 39]. The possible mechanism may attribute to the switching action of the AFP enhancer from the AFP promoter to the albumin promoter, which leads to a decrease in AFP expression and an increase in albumin expression [37]. Thus, in patients with chronic Hepatitis C, a reactive expression of the AFP gene, as shown in hepatic necroinflammation and hepatocellular proliferation, may be associated with a decrease in albumin gene transcription and may lead to a lower serum albumin level.

 The relationship between the level of aminotransferases and AFP is not definite as a rise in the level of aminotransferases enzymes can be an attribute to damage of hepatocytes, while the level of AFP, and especially a markedly increased level (>400 ng/mL), is rather due to a neoplasm, hepatocellular carcinoma [33]. In this study a negative correlation between the ALT, AST, and AFP (Table 2) was found but not statistically significant. Chu et al. found no significant different in serum transaminase levels in patients with or without elevated serum AFP [35], but against this result is that of Goldstein et al., who found that increasing serum AFP values were significantly correlated with increasing ALT values [8].

 There seems to be important correlations among the necroinflammation as well as the severity of fibrosis/cirrhosis and albumin with serum AFP. The necroinflammation as well as the severity of fibrosis/cirrhosis and a lower serum albumin was shown to be predictor for elevation of serum AFP among chronic HCV with steatosis.

 Our results were consistent with the report from Bayati et al., which revealed that an elevated serum AFP level was highly specific for the diagnosis of cirrhosis among patients with chronic hepatitis C. These findings indicated that hepatic fibrosis/cirrhosis is more important than necroinflammation in causing an elevation of serum AFP in patients with chronic hepatitis C [40]. Also chu et al. found that a lower serum albumin level was an independent factor in predicting elevated serum AFP [35].

In conclusion, this study has demonstrated that patients with chronic HCV and steatosis have higher AFP levels than those without steatosis. AFP correlates with necroinflammation as well as the severity of fibrosis/cirrhosis in chronic HCV with steatosis. Higher levels of serum AFP may correspond to the presence of steatosis among chronic HCV. They increased both the number of HPC and the extent of the ductular reaction in addition to increased fibrosis/cirrhosis levels providing a potential mechanism whereby steatosis contributes to the increase in AFP. So in the absence of traditional causes of elevated serum AFP, steatosis should be among the differential diagnoses of elevated serum AFP levels in patients with chronic HCV infection.

## Figures and Tables

**Table 1:** Clinical, biochemical and histopathological characteristics of patients with and without steatosis.

	Steatosis + VE	Steatosis − VE	*P* value
Age (years)	41.62 ± 6.37	43.68 ± 6.94	0.126
Grade (Ishak grading)	5.90 ± 2.21	2.48 ± .1.50	0.001*
Stage of fibrosis (Ishak staging)	2.72 ± 1.93	.50 ± .50	0.001*
AFP (ng/mL)	8.64 ± 3.18	3.56 ± .81	0.001*
Triglyceride (mg/dL)	108.95 ± 26.39	102.68 ± 31.65	0.323
FB glucose (mg/dL)	106.76 ± 75.84	101.31 ± 14.35	0.44
Alumin (g/dL)	4.40 ± .30	4.50 ± .28	0.115
AST (u/L)	53.62 ± 20.83	34.42 ± 15.26	0.001*
ALT (u/L)	57.72 ± 20.43	45.22 ± 18.47	0.002*
Total Bilirubin (mg/dL)	0.82 ± .35	0.62 ± .34	0.023*
Prothrombin time concentration %	88.09 ± 7.39	84.50 ± 3.64	0.003*
BMI	29.3 ± 5.2	28.9 ± 3.2	0.695
HCV RT-RNA (IU/mL)	224529.1 ± 308275.96	298415.9 ± 320004.30	0.302

AFP, Alpha-fetoprotein; FB glucose, Fasting blood glucose; AST, aspartate aminotransferase; ALT, alanine aminotransferase; BMI, body mass index; RT-PCR, reverse transcriptase-polymerase chain reaction.

**Table 2:** The Pearson's Correlation between pathological and biochemical parameters with AFP in CHC patients with steatosis.

	AFP	
	*R*	*P*
Necroinflammation	*0.759	0.001
Fibrosis	*0.759	0.001
Albumin (g/dL)	−0.710	0.001
ALT (u/L)	−0.041	0.778
AST (u/L)	−0.122	0.398

ALT, alanine aminotransferase; AST, aspartate aminotransferase.

## Abbreviations

AFP: Alpha-fetoprotein
CHC: Chronic hepatitis C
AST: Aspartate aminotransferase
ALT: Alanine aminotransferase
HCV: Hepatitis C virus
RT-PCR: Reverse transcriptase-polymerase chain reaction
BMI: Body mass index
HAI: Histological activity index.
